# Local electroneutrality breakdown for electrolytes within varying-section nanopores

**DOI:** 10.1140/epje/s10189-024-00408-9

**Published:** 2024-02-19

**Authors:** Paolo Malgaretti, Ignacio Pagonabarraga, Jens Harting

**Affiliations:** 1 Helmholtz Institute Erlangen-Nürnberg for Renewable Energy (IEK-11), Forschungszentrum Jülich, Erlangen, Germany; 2 Departament de Física de la Matèria Condensada, Universitat de Barcelona, Martí i Franqués 1, 08028 Barcelona, Spain; 3 Universitat de Barcelona Institute of Complex Systems (UBICS), Universitat de Barcelona, 08028 Barcelona, Spain; 4 Department of Chemical and Biological Engineering and Department of Physics, Friedrich-Alexander-Universität Erlangen-Nürnberg, Erlangen, Germany

## Abstract

**Abstract:**

We determine the local charge dynamics of a $z-z$ electrolyte embedded in a varying-section channel. By means of an expansion based on the length scale separation between the axial and transverse direction of the channel, we derive closed formulas for the local excess charge for both, dielectric and conducting walls, in 2*D* (planar geometry) as well as in 3*D* (cylindrical geometry). Our results show that, even at equilibrium, the local charge electroneutrality is broken whenever the section of the channel is not homogeneous for both dielectric and conducting walls as well as for 2*D* and 3*D* channels. Interestingly, even within our expansion, the local excess charge in the fluid can be comparable to the net charge on the walls. We critically discuss the onset of such local electroneutrality breakdown in particular with respect to the correction that it induces on the effective free energy profile experienced by tracer ions.

**Graphical abstract:**

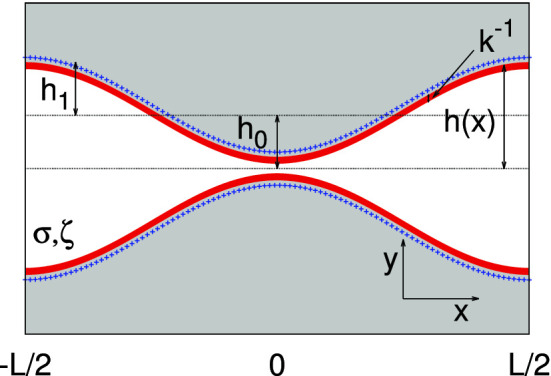

## Introduction

Understanding the dynamics of electrolytes embedded in varying section pores is crucial for many biological [1] as well as technological applications [2, 3]. For example, ion-channels [4], plant circulation [5], as well as lymphatic [6] and interstitial [7] transport rely on the active transport of electrolytes across tortuous conduits. Moreover, resistive-pulse sensing techniques measure tracer properties during their transport across charged nanopores [8–11] and energy-harvesting devices exploit the physical properties of confined electrolytes [12–14].

From a theoretical perspective, the usual approach is based on the Poisson–Boltzmann theory, within which the ions are regarded as point-like particles whose distribution fulfills the Boltzmann weight that is eventually determined by the solution of the Poisson equation. Such an approach can recover the dynamics of electrolytes confined between charged plates provided that the ions are sufficiently diluted and the surface charges are not too large. Within such conditions, the Poisson–Boltzmann theory recovers the global electroneutrality, namely the total charge in the liquid phase matches the one on the plates. However, recent experimental [15] and theoretical [16–19] results show that when the plates are approaching at distances $\lesssim 10$nm the reduced space and the steric and electrostatic interactions between the dissolved ions may lead to a breakdown of the global electroneutrality. This means that the net charge in the liquid between the plates does not balance the charge on the plates. However, since the electrolyte is typically in contact with a reservoir, the eventual global electroneutrality is attained when accounting for the charge distribution outside the channel [16, 20]. While global electroneutrality breakdown is associated with a significant increase in the total free energy of the system [21], local rearrangements of the charge may occur such that the electroneutrality is fulfilled globally but not locally, as it has been recently predicted for macroion solutions [22].

In particular, when the section of the confining vessel is not constant, novel dynamical regimes appear. Indeed, asymmetric pores have been used to rectify ionic currents [23–25], as well as to realize highly sensitive dopamine-responsive iontronic devices [26]. Moreover, recirculation and local electroneutrality breakdown have been reported for electrolytes confined between corrugated walls [27, 28] and the variation in channel section can tune their permeability [29, 30] and even enhance the effective transport coefficients [31].

Accordingly, the question arises about the interplay between the geometry of the pore and the local electroneutrality breakdown. In order to be able to efficiently explore the parameter space we aim at an analytical approach that can provide closed formulas capturing the dependence of electroneutrality breakdown on the parameters characterizing the system. Accordingly, we exploit the (linearized) Poisson–Boltzmann theory. Interestingly, our results show that even in the simplest scenario, i.e., at equilibrium and within the Debye-Hückel approximation, the interplay between the (varying) local section of the channel and the electrostatic forces leads to a breakdown of local electroneutrality and to the onset of an inhomogeneous excess charge for both dielectric and conducting channel walls in planar (2*D*) and cylindrical (3*D*) geometries. Interestingly, upon tuning the parameters, the local excess charge can be as large as the local charge on the walls. Once integrated along the transverse direction the local excess charge can be expressed via a multipole expansion whose leading term is a quadrupole. Finally, such an insight can be used to predict the corrections to the effective free energy profile experienced by a tracer ion induced by the local excess charge.

## 2*D* model

**Fig. 1:**
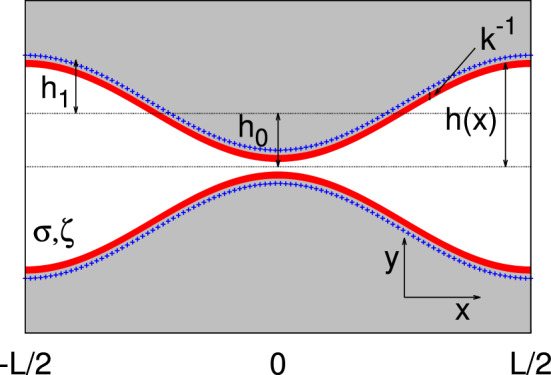
Schematic view of the system: channel walls are in grey, the local surface charge is represented by blue crosses whereas the width of the Debye double layer is in red

In the following, we analyze the case of a channel whose half-section *h*(*x*) (see Fig. 1)

1 $$\begin{aligned} h(x)=h_0\left( 1-h_1\cos (2\pi x/L)\right) \end{aligned}$$

varies solely along the *x* direction. For later use, we introduce the entropic barrier [32–34]

2 $$\begin{aligned} \Delta S=\ln \left[ \frac{h_{\text {max}}}{h_\text {min}}\right] . \end{aligned}$$

Here, $h_{\text {max}}\equiv h_0(1+h_1)$ and $h_\text {min}\equiv h_0(1-h_1)$ are, respectively, the maximum and minimum channel sections. The electrostatic potential $\phi$ inside the channel is determined by solving the (linearized) Poisson–Boltzmann equation,

3 $$\begin{aligned} \nabla _{x,y}^{2}\phi (\textbf{x})=k^2 \phi (\textbf{x}), \end{aligned}$$

where $k^{-1}=\lambda$ is the inverse Debye length.

In order to get an analytical insight into the solution of Eq. (3) we exploit the lubrication approximation, based on a separation between longitudinal and transverse length scales. In the system under study, the longitudinal length scale is captured by *L* which is the period of the channel section, whereas the transverse length scales are the channel average section $h_0$ and the Debye length $\lambda$. In typical situations we have that $L\gg \lambda$ and hence the Laplace operator can be simplified by assuming that the changes of $\phi$ along the longitudinal direction are much smaller than those along the transverse direction. Hence, we identify $\lambda /L$ as the small parameter. Accordingly, we rewrite Eq. (3) as

4 $$\begin{aligned} \phi = \frac{\lambda ^2}{L^2} \frac{\partial ^2 \phi }{\partial ^2 x_*}+\frac{\partial ^2 \phi }{\partial y_*^{2}}{\simeq \frac{\partial ^2 \phi }{\partial y_*^{2}}}. \end{aligned}$$

In the last expression, we have highlighted that the first term on the left-hand side is of $\mathcal {O}\left( \frac{\lambda }{L}\right) ^2$ as compared to the second one. Accordingly, in the lubrication approximation, we treat the dependence on the longitudinal coordinate parametrically.

The solution of Eq. (3) is governed by the boundary conditions on the channel walls that in the following are assumed to be the same on both walls. For conducting channel walls the potential equals the zeta potential $\zeta$:

5 $$\begin{aligned} \phi _0(x,{\pm }h(x))=\zeta \, \end{aligned}$$

Alternatively, for insulating channel walls, we have that

6 $$\begin{aligned} -\nabla \phi \cdot \textbf{n}|_{y=\pm h(x)}= & {} \textbf{E}\cdot \textbf{n}=\pm \frac{\sigma }{\epsilon }, \end{aligned}$$

where $\textbf{n}$ is the unit vector normal at the channel walls and pointing inside the channel.

In particular, calling $\alpha =\arctan (\partial _xh(x))$ the local slope of the channel walls and focusing on the upper channel wall (the same calculations can be re-derived for the lower one) we have:

7 $$\begin{aligned} \textbf{n}= & {} \left( \begin{array}{c} \sin \alpha \\ -\cos \alpha \end{array}\right) \end{aligned}$$

Accordingly, we get

8 $$\begin{aligned} \left[ -\partial _x\phi (x,y)\sin \alpha +\partial _y\phi (x,y)\cos \alpha \right] _{y=\pm h(x)}&=\pm \frac{\sigma }{\epsilon }. \end{aligned}$$

In order to determine the boundary condition at different orders in the lubrication expansion we recall that

9 $$\begin{aligned} \alpha \simeq \partial _x h(x) +\mathcal {O}\left( \frac{h_0}{L}\right) ^3 \end{aligned}$$

and hence we have that at leading order in lubrication the boundary condition reads

10 $$\begin{aligned} \partial _y \phi _0(x,y)|_{y=\pm h(x)}=\pm \frac{\sigma }{\epsilon }, \end{aligned}$$

whereas at the second order (i.e. next leading order) we have

11 $$\begin{aligned} \partial _y&\phi _2(x,y)|_{y=\pm h(x)}\nonumber \\&\!\!\!=\left[ \frac{1}{2}\partial _y \phi _0(x,y)(\partial _x h(x))^2+\partial _x\phi _0(x,y)\partial _x h(x)\right] _{y=\pm h(x)}. \end{aligned}$$

At equilibrium, we use the following *ansatz* for the linearized ionic number densities:

12a $$\begin{aligned} \tilde{\rho }_{+}(x,y)= & {} \rho _{+}(x)\left( 1-\beta ez\phi (x,y)\right) \end{aligned}$$

12b $$\begin{aligned} \tilde{\rho }_{-}(x,y)= & {} \rho _{-}(x)\left( 1+\beta ez\phi (x,y)\right) \end{aligned}$$

## 2*D* first order lubrication approximation

At leading order in the lubrication expansion, namely at order $\mathcal {O}(h_0/L)^0$, the ionic number densities read

13a $$\begin{aligned} \tilde{\rho }_{+,0}(x,y)= & {} \rho _{+,0}(x)\left( 1-\beta ez\phi _0(x,y)\right) , \end{aligned}$$

13b $$\begin{aligned} \tilde{\rho }_{-,0}(x,y)= & {} \rho _{-,0}(x)\left( 1+\beta ez\phi _0(x,y)\right) \end{aligned}$$

and the Poisson equation reads

14 $$\begin{aligned} \partial _{y}^{2}\phi _0(x,y)= -\frac{ze}{\epsilon }\left[ \psi _0(x)-\varphi _0(x)\beta ze\phi _0(x,y)\right] , \end{aligned}$$

where we have introduced the notation

15a $$\begin{aligned} \varphi _0(x)&=\rho _{+,0}(x)+\rho _{-,0}(x), \end{aligned}$$

15b $$\begin{aligned} \psi _0(x)&=\rho _{+,0}(x)-\rho _{-,0}(x). \end{aligned}$$

The solution of Eq. (14) is then given by

16 $$\begin{aligned} \phi _0(x,y)=A_0(x)\cosh \left( k_0(x)y\right) +\frac{ze}{\epsilon k_0^{2}(x)}\psi _0(x), \end{aligned}$$

where

17 $$\begin{aligned} k_0(x)=\sqrt{\frac{\beta (ze)^2}{\epsilon }\varphi _0(x)} \end{aligned}$$

is the, zeroth-order, inverse Debye length and $A_0$ is determined by the boundary conditions imposed by the channel walls. For conducting channel walls the potential equals the zeta potential $\zeta$ at the walls, $\phi _0(x,h(x))=\zeta$. Hence, we have

18 $$\begin{aligned} \phi _0(x,y)&=\zeta \frac{\cosh \left( k_0(x)y\right) }{\cosh \left( k_0(x)h(x)\right) }\nonumber \\&\quad +\frac{ze}{\epsilon k^{2}}\psi _0(x)\left( 1-\frac{\cosh \left( k_0(x)y\right) }{\cosh \left( k_0(x)h(x)\right) }\right) . \end{aligned}$$

Alternatively, for insulating channel walls and for smoothly varying-channels, $\partial _x h(x)\ll 1$, the boundary condition can be expressed as [29]

19 $$\begin{aligned} \left. \frac{\partial \phi _0}{\partial y}\right| _{y=\pm h(x)}=\pm \frac{\sigma }{\epsilon }\left( 1+\frac{1}{2}\left( \partial _xh(x)\right) ^2\right) +\mathcal {O}(\partial _x h(x))^3, \end{aligned}$$

where we have substituted $\alpha \simeq \partial _xh(x)$ in Eq. (19). Accordingly, using Eq. (19) the electrostatic potential reads

20 $$\begin{aligned} \phi _0(x,y)=\frac{\sigma }{\epsilon k_0(x)}\frac{\cosh \left( k_0(x)y\right) }{\sinh \left( k_0(x)h(x)\right) }+\frac{ze}{\epsilon k_0^{2}(x)}\psi _0(x)\,. \end{aligned}$$

At equilibrium, in order to solve for $\rho _{+,0}(x),\rho _{-,0}(x)$, we impose that the electrochemical potential is constant [35]:

21 $$\begin{aligned} \mu _\pm (x,y)=k_BT \ln \tilde{\rho }_\pm (x,y) \pm ze\phi (x,y)=\bar{\mu }_\pm . \end{aligned}$$

Disregarding terms of order $\mathcal {O}(\phi _0)^2$, by plugging Eq. (13) into Eq. (21) leads to

22 $$\begin{aligned} \rho _{\pm ,0}(x)=e^{\beta \bar{\mu }_\pm } \end{aligned}$$

and the constant values of $\rho _{\pm ,0}$ are set by the equilibrium chemical potentials $\bar{\mu }_\pm$. For $z-z$ electroneutral systems we have $\mu _+=\mu _-$ and therefore

23 $$\begin{aligned} \rho _{+,0}=\rho _{-,0}\,. \end{aligned}$$

This implies

24 $$\begin{aligned} \varphi _0(x)&=2\rho _0, \end{aligned}$$

25 $$\begin{aligned} \psi _0(x)&=0. \end{aligned}$$

In particular, Eq. (24) leads to

26 $$\begin{aligned} k_0(x)=k_0, \end{aligned}$$

i.e., the Debye length is constant along the channel. Finally, the electrostatic potential for conducting channel walls reads

27 $$\begin{aligned} \phi ^\zeta _0(x,y)=\zeta \frac{\cosh \left( k_0y\right) }{\cosh \left( k_0h(x)\right) }, \end{aligned}$$

whereas for insulating walls it is

28 $$\begin{aligned} \phi ^\sigma _0(x,y)=\frac{\sigma }{\epsilon k_0}\frac{\cosh \left( k_0y\right) }{\sinh \left( k_0h(x)\right) }. \end{aligned}$$

Substituting Eq. (27) or Eq. (28) into Eq. (14) and using Eq. (25) the net local charge reads, respectively,

29 $$\begin{aligned}&q^\zeta _0(x)=-\!\!\!\!\int \limits _{-h(x)}^{h(x)}\!\!\!\epsilon k_0^2 \zeta \frac{\cosh \left( k_0y\right) }{\cosh \left( k_0h(x)\right) }dy =-2\epsilon k_0 \zeta \tanh (k_0 h(x)), \end{aligned}$$

30 $$\begin{aligned}&q^\sigma _0(x)=-\!\!\!\!\int \limits _{-h(x)}^{h(x)}\!\!\!\epsilon k_0^2 \frac{\sigma }{\epsilon k_0}\frac{\cosh \left( k_0y\right) }{\sinh \left( k_0h(x)\right) }dy=-2\sigma , \end{aligned}$$

which recovers the local electroneutrality of the system.^1^ Equations (26)–(30) show that at leading order in lubrication, there is no correction to the Debye length, electrostatic potential or local charge and ionic density induced by the geometrical confinement. Such a tight relationship between the surface charge and the charge in the liquid phase is carved into the Debye-Hückel equation. In fact, multiplying by $-\epsilon$ and integrating the Debye-Hückel equation at first order in lubrication along the transverse coordinate leads to

31 $$\begin{aligned} -\epsilon \int _{-h(x)}^{h(x)}\partial ^2_y \phi (x,y) dy = -\epsilon \int _{-h(x)}^{h(x)}k_0^2 \phi (x,y) dy. \end{aligned}$$

After integrating it follows that

32 $$\begin{aligned} -\epsilon \partial _y \phi (x,y)|^{h(x)}_{-h(x)}=2\sigma =q(x), \end{aligned}$$

where we have identified that the right-hand side of Eq. (31) is indeed the total charge in the fluid and the left-hand side corresponds to minus the surface charge. Therefore, in order to check if local electroneutrality can be broken also at equilibrium, we need to account for higher order corrections in the lubrication approximation.

## 2*D* higher order corrections

It is clear that, at order $\mathcal {O}(\frac{h_0}{L})$, Eq. (4) jointly with the boundary conditions, Eq. (19) leads to $\phi _1(x,y)=0$. Therefore, the next leading order is $\mathcal {O}(\frac{h_0}{L})^2$. Accordingly, the ansatz of the ionic number densities reads

33a $$\begin{aligned} \tilde{\rho }_{+,2}(x,y)&= \rho _{+,2}(x)\left( 1-\beta ez\phi _0(x,y)\right) -\rho _{+,0}\beta ez\phi _2(x,y), \end{aligned}$$

33b $$\begin{aligned} \tilde{\rho }_{-,2}(x,y)&= \rho _{-,2}(x)\left( 1+\beta ez\phi _0(x,y)\right) +\rho _{-,0}\beta ez\phi _2(x,y). \end{aligned}$$

At equilibrium the chemical potential is homogeneous. Therefore, since the zeroth order contribution to the chemical potential is already fulfilling the equilibrium conditions, see Eq. (21), we have that

34 $$\begin{aligned} \mu _{\pm ,2}(x,y)=0. \end{aligned}$$

The expression for the second order contribution to the local chemical potential reads

35 $$\begin{aligned} \mu _{\pm ,2}(x,y)=k_B T\frac{\tilde{\rho }_{\pm ,2}(x,y)}{\rho _{\pm ,0}}\pm ze \phi _2(x,y). \end{aligned}$$

Using Eqs. (22),(23),(33a),(33b) and keeping only terms linear in the electrostatic potential, Eq. (34) leads to

36a $$\begin{aligned} \rho _{+,2}(x)\left( 1-\beta ze\phi _0(x,y)\right)&=0, \end{aligned}$$

36b $$\begin{aligned} \rho _{-,2}(x)\left( 1+\beta ze\phi _0(x,y)\right)&=0, \end{aligned}$$

from which we obtain

37a $$\begin{aligned} \rho _{+,2}(x)&=0, \end{aligned}$$

37b $$\begin{aligned} \rho _{-,2}(x)&=0. \end{aligned}$$

Accordingly, the Poisson equation reads

38 $$\begin{aligned} \partial _{x}^{2}\phi _0(x,y)+\partial _{y}^{2}\phi _2(x,y)= k_0^2\phi _2(x,y)\,. \end{aligned}$$

We remark that, due to Eq. (37), there is no second order correction to the Debye length, which indeed is clear from Eq. (38). The solution of Eq. (38), using Eq. (16), reads

39 $$\begin{aligned} \phi&_2(x,y)=A_2(x)\cosh (k_0 y)+\frac{1}{4k_0^2}\partial ^2_x A_0(x)\xi (x,y), \end{aligned}$$

with

40 $$\begin{aligned} \xi (x,y)=\cosh (k_0 y)-2k_0 y\sinh (k_0 y), \end{aligned}$$

where $A_0(x)$ is the zeroth–order integration constant and it is determined by the following boundary conditions:

41 $$\begin{aligned} A^\zeta _0(x)=\frac{\zeta }{\cosh (k_0 h(x))} \end{aligned}$$

for conducting walls and

42 $$\begin{aligned} A^\sigma _0(x)=\frac{\sigma }{\epsilon k_0}\frac{1}{\sinh (k_0 h(x))} \end{aligned}$$

for dielectric walls. Finally, $\phi _{2}(x,y)$ is determined by imposing the boundary conditions at the channel walls. For conducting channel walls, at order $\mathcal {O}\left( \frac{h_0}{L}\right) ^2$, the boundary condition reads

43 $$\begin{aligned} \phi _2(x,\pm h(x))=0. \end{aligned}$$

This leads to

44 $$\begin{aligned} \!\phi ^\zeta _2(x,y)=\!\frac{\partial ^2_x A^\zeta _0(x)}{4k_0^2}\left[ \xi (x,y)-\frac{\cosh (k_0 y)}{\cosh (k_0h(x))}\xi (x,h(x))\right] . \end{aligned}$$

In contrast, for dielectric channel walls the boundary conditions, at order $\mathcal {O}(\frac{h_0}{L})^2$ is given by Eq. (19) which leads to (similar results can be obtained for $y=-h(x)$)

45 $$\begin{aligned} \partial _y\phi _2|_{y=h(x)}=&\frac{1}{2}\frac{\sigma }{\epsilon }\left( \partial _xh(x)\right) ^2\nonumber \\&+\cosh (k_0 h(x))\partial _x A_0(x)\partial _x h(x), \end{aligned}$$

from which we obtain

46 $$\begin{aligned} A^\sigma _2(x)&= \frac{\sigma }{2 k \epsilon }\frac{\left( \partial _xh(x)\right) ^2}{\sinh (k_0 h(x))}\nonumber \\&\quad +\frac{\cosh (k_0 h(x))}{k_0\sinh (k_0 h(x))}\partial _x A^\sigma _0(x)\partial _x h(x)\nonumber \\&\quad +\frac{\partial ^2_x A^\sigma _0(x)}{4k_0^2}\left[ 1+2k_0 h(x)\frac{\cosh (k_0 h(x))}{\sinh (k_0 h(x))}\right] \end{aligned}$$

and accordingly we get

47 $$\begin{aligned}&\phi ^\sigma _2(x,y)=\frac{1}{2}\phi ^\sigma _0(x,y)\left( \partial _x h(x)\right) ^2\nonumber \\&\quad +\frac{\epsilon }{\sigma }\phi ^\sigma _0(x,y)\cosh (k_0h(x))\partial _x A^\sigma _0(x)\partial _x h(x) \nonumber \\&\quad +\frac{\partial ^2_x A^\sigma _0(x)}{2k_0^2}\left[ \left( k_0h(x)\frac{\cosh (k_0 h(x))}{\sinh (k_0h(x))} +1\right) \cosh (k_0 y)\right. \nonumber \\&\quad \left. -k_0y \sinh (k_0 y)\right] . \end{aligned}$$

## 2*D* local electroneutrality breakdown

*a. Dielectric channel walls* Using Eq. (47) we can calculate the corrections to the local charge for dielectric channel walls^2^:

48 $$\begin{aligned} q_2^\sigma (x)&=-\epsilon k_0^2 \int _{-h(x)}^{h(x)}\phi ^\sigma _2(x,y)dy\nonumber \\&= -\sigma \left( \partial _x h(x)\right) ^2 - 2\epsilon \cosh (k_0h(x))\partial _x A^\sigma _0(x)\partial _x h(x) \nonumber \\&\quad -2\epsilon \frac{\partial ^2_x A^\sigma _0(x)}{k_0}\sinh (k_0 h(x)) \end{aligned}$$

For a dielectric channel, at second order in lubrication $\mathcal {O}(h_0/L)^2$, the charge per unit area on the channel walls reads

49 $$\begin{aligned} q^\sigma _w(x)=\sigma \left( 1+\frac{1}{2}\left( \partial _x h(x)\right) ^2\right) . \end{aligned}$$

Accordingly, we can define the excess charge

50 $$\begin{aligned} \Delta q^\sigma (x)&=\frac{q^\sigma _0+q^\sigma _2(x)+2q^\sigma _w(x)}{2 |q_{0}^\sigma |}. \end{aligned}$$

Using Eqs. (30),(48),(49), at second order in lubrication, the last expression reduces to

51 $$\begin{aligned} \Delta q^\sigma (x)\simeq&-\frac{\epsilon }{k_0 \sigma } \left[ \partial _x\sinh (k_0h(x)) \partial _x A^\sigma _0(x)\right. \nonumber \\&\left. +\partial ^2_x A^\sigma _0(x)\sinh (k_0 h(x))\right] \,. \end{aligned}$$

This can be rewritten as

52 $$\begin{aligned} \Delta q^\sigma (x)\simeq - \frac{\epsilon }{k_0 \sigma }\partial _x\left[ \sinh (k_0 h(x))\partial _x A_0^\sigma (x)\right] \,. \end{aligned}$$

**Fig. 2:**
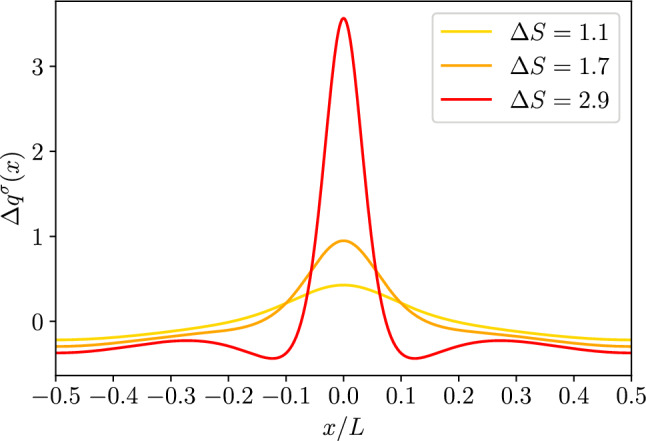
2*D* dielectric channel ($\sigma >0$). Local excess charge $\Delta q(x)$ as a function of the normalized position *x*/*L* along the channel axis for different values of $\Delta S$ as detailed in the legend with $h_0/L=0.1$, and $kh_0=1$

Figure 2 shows the dependence of $\Delta q^\sigma$ on the longitudinal position. Interestingly, there is a depletion (with respect to the case of planar channel walls) of counterions close to the bottleneck, at $x/L={0}$, that leads to a net local positive charge. In order to fulfill global electroneutrality, this is compensated for by a net excess of counterions in the rest of the channel. As expected due to symmetry, the distribution of the excess charge is symmetric about the center of the channel and, in a far–field expansion, it leads to the onset of a net quadrupolar contribution.

**Fig. 3:**
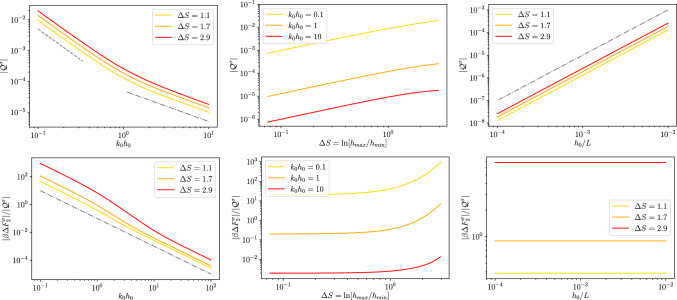
2*D* dielectric channel ($\sigma >0$). Top left: absolute value of the quadrupole moment, as defined in Eq. (53), $\mathcal {Q}^\sigma$ as a function of $k_0 h_0$ for different values of $\Delta S$ as reported in the legend and with $h_0/L=0.01$. The grey dashed line is proportional to $\propto (k_0 h_0)^{-1}$ and the dashed-dotted one to $\propto (k_0 h_0)^{-2}$. Top center: absolute value of the quadrupole moment, $\mathcal {Q}^\sigma$, as a function of $\Delta S$ for different magnitudes of $kh_0$ as reported in the legend and with $h_0/L=0.1$. The thin grey dotted line is a guide for the eye and it is proportional to $\Delta S^2$. Top right: absolute value of the quadrupole momentum, $\mathcal {Q}^\sigma$, as a function of $h_0/L$ for different magnitudes of $\Delta S$ as reported in the legend and with $kh_0 = 1$. The thin grey dotted line is a guide for the eye and it is proportional to $(h_0/L)^2$. Bottom: ratio $\Delta F^\sigma _2/\mathcal {Q}^\sigma$ for the same values of the parameters of the corresponding top panel

We remark that when the local excess charge $\Delta q^\sigma (x)$ is integrated over the channel period it leads to a vanishing contribution, i.e., global electroneutrality is retrieved. We quantify the magnitude of the local electroneutrality breakdown by computing the dimensionless quadrupolar moment

53 $$\begin{aligned} \mathcal {Q}^\sigma = \frac{1}{L}\int _{-\frac{L}{2}}^{\frac{L}{2}}\frac{x^2}{L^2}\Delta q^\sigma (x) dx. \end{aligned}$$

The top panel of Fig. 3 shows the dependence of $\mathcal {Q}^\sigma$ on $k_0 h_0$. Interestingly, $\mathcal {Q}^\sigma$ shows a twofold scaling: for smaller values of $k_0 h_0$, $\mathcal {Q}^\sigma$ decays as $k_0 h_0^{-2}$, whereas for larger values of $k_0 h_0$ it decays as $k_0 h_0^{-1}$. In particular, the crossover between the two regimes is around $k_0 h_0\simeq 1$, i.e., when the Debye length is comparable to the average channel section. The central panel of Fig. 3 shows that $\mathcal {Q}^\sigma$ grows almost linearly with the entropic barrier $\Delta S$. Finally, the bottom panel of Fig. 3 shows that $\mathcal {Q}^\sigma$ depends quadratically on $h_0/L$. This is expected since these results were derived at the second order in lubrication.

*b. Conducting channel walls* Using Eq. (44) we can calculate the corrections to the local charge for conducting channel walls^3^:

54 $$\begin{aligned} q_2^\zeta (x)&=-\epsilon k_0^2 \int _{-h(x)}^{h(x)}\phi ^\zeta _2(x,y)dy\nonumber \\&=\epsilon \frac{\partial ^2_x A^\zeta _0(x)}{k_0}\left[ \frac{k_0 h(x)}{\cosh (k_0 h(x)}-\sinh (k_0 h(x))\right] \end{aligned}$$

For a conducting channel, we first define the effective surface charge as

55 $$\begin{aligned} q_w^\zeta (x) \equiv -\epsilon \nabla \phi ^\zeta (x,y)|_{y=h(x)}\cdot \textbf{n}, \end{aligned}$$

which up to second order contributions in lubrication reads as

56 $$\begin{aligned} q_w^\zeta (x)&\simeq -\epsilon \left[ \partial _x \phi _0^\zeta (x,y)\partial _x h(x)\right. \nonumber \\&\quad \left. -\partial _y\phi _0(x,y)\left( 1-\frac{1}{2}(\partial _xh(x))^2\right) -\partial _y\phi _2(x,y)\right] _{y=h(x)}. \end{aligned}$$

This can be rewritten as

57 $$\begin{aligned} q_w^\zeta (x) \simeq&-\epsilon \left[ \cosh (k_0 h(x))\partial _x A_0^\zeta (x) \partial _x h(x)\right. \nonumber \\&-\zeta k_0 \text {tanh}(k_0 h(x))\left( 1-\frac{1}{2}(\partial _xh(x))^2\right) \nonumber \\&\left. +\frac{\partial _x^2 A_0^\zeta (x)}{2 k_0}\left( \frac{k_0 h(x)}{\cosh (k_0 h(x)}+\sinh (k_0 h(x))\right) \right] . \end{aligned}$$

We recall that the last expression accounts for the surface charge density along the surface of the channel. However, when comparing the surface charge to that in the liquid phase we have to account for the fact that the latter is per unit length *dx* along the longitudinal axis of the channel. Accordingly, when computing the local electroneutrality we have to multiply Eq. (57) by the local area $\simeq 1+\frac{1}{2}(\partial _x h(x))^2$. We then get

58 $$\begin{aligned} q_w^\zeta (x)&\simeq \epsilon \left[ \zeta k_0 \text {tanh}(k_0 h(x))-\cosh (k_0 h(x))\partial _x A_0^\zeta (x) \partial _x h(x)\right. \nonumber \\&\quad \left. -\frac{\partial _x^2 A_0^\zeta (x)}{2 k_0}\left( \frac{k_0 h(x)}{\cosh (k_0 h(x)}+\sinh (k_0 h(x))\right) \right] . \end{aligned}$$

We define the excess charge as

59 $$\begin{aligned} \Delta q^\zeta (x)&=\frac{q^\zeta _0+q^\zeta _2(x)+2q^\zeta _w(x)}{2 |q^\zeta _{0}|}, \end{aligned}$$

In this case, we chose a different normalization as compared to the dielectric case because at zeroth order in lubrication, the local surface charge is not homogeneous and this would lead to unphysical contribution when assessing the global electroneutrality. Finally, using Eqs. (29), (54), (58) and (59) reduces to

60 $$\begin{aligned} \Delta q^\zeta (x)&\simeq -\dfrac{\partial _x\left[ \partial _x A_0^\zeta (x)\sinh (k_0 h(x))) \right] }{k_0^2\zeta \text {tanh}(k_0 h_0)} \,. \end{aligned}$$

**Fig. 4:**
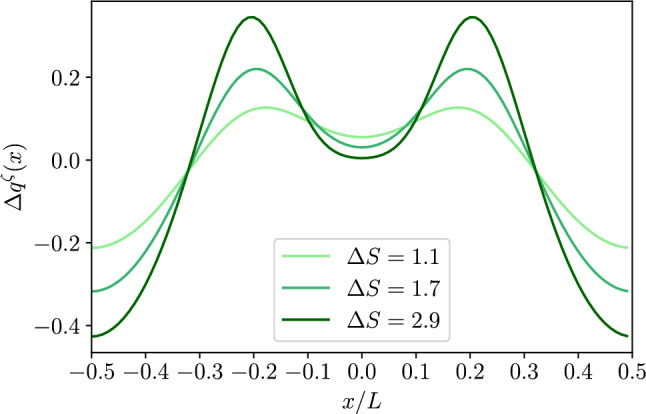
2*D* conducting channel ($\zeta >0$). Local excess charge $\Delta q(x)$ as a function of the normalized position *x*/*L* along the channel axis for different values of $\Delta S$ as deailed in the legend with $h_0/L=0.1$ and $kh_0=1$

As for the dielectric case, we note that Eq. (60) shows the onset of local electroneutrality breakdown but also the fulfillment of global electroneutrality once $\Delta q^\zeta$ is integrated over the channel period. By comparing Fig. 2 with Fig. 4 we note that while for the dielectric channel there is a clear and sharp peak at the channel bottleneck, for the conducting channel the peaks are located where the slope of the channel walls is maximum, i.e., $x/L=\pm 0.25$. At the same time, on the top of the shift of the maxima, we also observe that the magnitude of the peak is reduced in the case of conducting as compared to dielectric channel walls. This may be due to the fact that, even at zeroth order in lubrication, the local surface charge density on the channel walls is not constant: Eq. (29) indeed shows that, for $k_0 h_0\simeq 1$, the local charge density is minimum at the channel bottleneck. Accordingly, a smaller local surface charge leads to a minor departure from local electroneutrality.

**Fig. 5:**
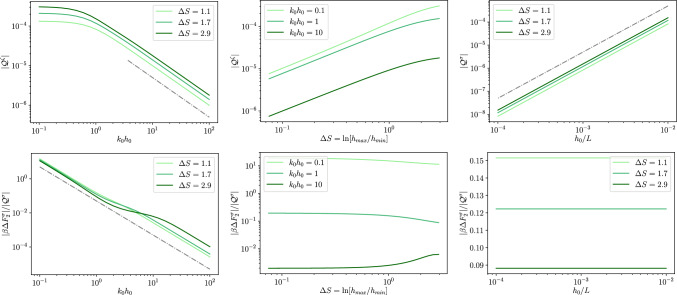
2*D* conducting channel ($\zeta >0$). Top left: absolute value of the quadrupole moment as defined in Eq. (53), $\mathcal {Q}^\sigma$ as a function of $k_0 h_0$ for different values of $\Delta S$ as reported in the legend and with $h_0/L=0.01$. The grey dashed line is proportional to $\propto (k_0 h_0)^{-1}$ and the dashed dotted to $\propto (k_0 h_0)^{-2}$. Top center: absolute value of the quadrupole moment, $\mathcal {Q}^\sigma$, as a function of $\Delta S$ for different magnitudes of $kh_0$ as reported in the legend and with $h_0/L=0.1$. The thin grey dotted line is a guide for the eye and it is proportional to $\Delta S^2$. Top right: absolute value of the quadrupole moment, $\mathcal {Q}^\sigma$, as a function of $h_0/L$ for different magnitudes of $\Delta S$ as reported in the legend and with $kh_0 = 1$. The thin grey dotted line is a guide for the eye and it is proportional to $(h_0/L)^2$. Bottom: ratio $\Delta F^\sigma _2/\mathcal {Q}^\sigma$ for the same values of the parameters of the corresponding top panel

For what concerns the magnitude of the local excess charge captured by the quadrupolar moment, the top panel of Fig. 5 shows that $\mathcal {Q}^\zeta$ decays as $1/k_0 h_0$ for larger values of $k_0 h_0$ (as it is for dielectric channel walls) whereas for $k_0 h_0 \ll 1$, at variance with dielectric walls, $\mathcal {Q}^\zeta$ attains a plateau. Finally, the central and bottom panels of Fig. 5 show a behaviour similar to that observed for dielectric channel walls.

## 2*D* free energy barrier

While the leading term for the local electroneutrality breakdown is quadratic in $h_0/L$ this is not the case for the equilibrium free energy profile. For a tracer ion with elementary charge *e*, the local free energy can be obtained from the logarithm of the partition function *Z*(*x*)

61 $$\begin{aligned} \beta F(x) = -\ln Z(x) = - \ln \left[ \frac{1}{2h_0}\int \limits _{-h(x)}^{h(x)}e^{-\beta e \phi (x,y)}dy\right] \end{aligned}$$

and within the Debye-Hückel approximation it becomes

62 $$\begin{aligned} \beta F(x) \simeq \beta F_{DH}(x) = -\ln \left[ \frac{1}{2h_0}\int \limits _{-h(x)}^{h(x)}1-\beta e\phi (x,y)dy\right] . \end{aligned}$$

By expanding the logarithm, Eq. (62) can be decomposed into the following contributions:

63 $$\begin{aligned} \beta F_{DH}(x) \simeq \beta F_{gas}(x)+\beta F_{0}(x)+\beta F_{2}(x), \end{aligned}$$

with

64 $$\begin{aligned} \beta F_{gas}(x)&= -\ln \left[ \frac{h(x)}{h_0}\right] , \end{aligned}$$

65 $$\begin{aligned} \beta F_0(x)&= \frac{\beta e}{2h(x)}\int \limits _{-h(x)}^{h(x)}\phi _0(x,y)dy=-\frac{\beta e q_0(x)}{2h(x)\epsilon k_0^2}, \end{aligned}$$

66 $$\begin{aligned} \beta F_2(x)&= \frac{\beta e}{2h(x)}\int \limits _{-h(x)}^{h(x)}\phi _2(x,y)dy=-\frac{\beta e q_2(x)}{2h(x)\epsilon k_0^2}, \end{aligned}$$

where $\beta F_{gas}(x)$ is the free energy profile of an uncharged point particle, whereas $\beta F_0(x)$ and $\beta F_2(x)$ are, respectively, the leading order and higher order correction for charged particles. The impact of the local excess charge on the dynamics of a tracer ion can be captured by the correction to the effective free energy barrier induced by the local excess charge

67 $$\begin{aligned} \Delta F_2 = F_2(L/2)-F_2(0). \end{aligned}$$

In particular, we are interested in quantifying the contribution of the quadrupole moment to such a correction to the free energy barrier. The bottom rows of Figs. 3 and 5 report the ratio between the second-order correction to the free energy difference and the quadrupole moment. As shown in the figures, the ratio of $\Delta F_2$ and $\mathcal {Q}$ is generally sensitive to both $k_0 h_0$ and $\Delta S$ hence highlighting the relevance of higher-order multipoles in the free energy difference. Finally, as expected $\Delta F_2$ and $\mathcal {Q}$ have the same scaling with $h_0/L$ and hence their ratio is insensitive to it.

## 3*D* first order lubrication approximation

Exploiting the experience gathered for the 2*D* case we can straightforward write down the solution of the Debye-Hückel equation in the case of axially symmetric channels which, at first order in lubrication, reads

68 $$\begin{aligned} \partial ^2_r \phi _0(x,r)+\frac{1}{r}\partial _r\phi _0(x,r)=k_0^2 \phi _0(x,r)\,. \end{aligned}$$

The solutions to it are

69 $$\begin{aligned} \phi ^\zeta _0(x,r)&= \zeta \frac{I_0(k_0r)}{I_0(k_0 h(x))}, \end{aligned}$$

70 $$\begin{aligned} \phi ^\sigma _0(x,r)&= \frac{\sigma }{\epsilon k}\frac{I_0(k_0r)}{I_1(k_0 h(x))}, \end{aligned}$$

where $I_n$ are modified Bessel functions of the first kind of order *n*. As for the 2*D* case, at linear order in the lubrication approximation the solution of the Debye-Hückel equation, Eq. (70), fulfills local electroneutrality:

71 $$\begin{aligned} q^\zeta _0(x)&= -2\pi \epsilon k_0^2 \int \limits _0^{h(x)} \phi ^\zeta _0(x,r) r dr = -2\pi \epsilon \zeta \frac{k_0 h(x) I_1(k_0 h(x))}{I_0(k_0 h(x))}, \end{aligned}$$

72 $$\begin{aligned} q^\sigma _0(x)&= -2\pi \epsilon k_0^2 \int \limits _0^{h(x)} \phi ^\sigma _0(x,r) r dr = -2\pi h(x)\sigma \,. \end{aligned}$$

This indeed is minus the charge on the wall.

## 3*D* higher order corrections

At second order in lubrication the Debye-Hückel equation becomes

73 $$\begin{aligned} \partial ^2_x \phi _0(x,r)+\partial ^2_r \phi _2(x,r)+\frac{1}{r}\partial _r\phi _2(x,r)=k_0^2 \phi _2(x,r)\,, \end{aligned}$$

whose solution reads^4^

74 $$\begin{aligned} \phi _2(x,r) = B_2(x)I_0(k_0 r)-\frac{\partial _x^2 B_0 (x)}{2k_0^2} \left( I_0(k_0r)+k_0rI_1(k_0 r)\right) , \end{aligned}$$

with

75 $$\begin{aligned} B^\zeta _0(x)&= \frac{\sigma }{\epsilon k_0}\frac{1}{I_0(k_0 h(x))}, \end{aligned}$$

76 $$\begin{aligned} B^\sigma _0(x)&= \frac{\sigma }{\epsilon k_0}\frac{1}{I_1(k_0 h(x))} \end{aligned}$$

and $B_2$ is determined by the boundary conditions.

## 3*D* local electroneutrality breakdown

*a. Dielectric channel walls* For dielectric channel walls the boundary condition is the same as the one derived for the 2*D* case, Eq. (19), and reads

77 $$\begin{aligned} \partial _r \phi ^\sigma _2(x,r)&= \frac{1}{2}\frac{\sigma }{\epsilon }(\partial _x h(x))^2\nonumber \\&\quad -\frac{\sigma }{\epsilon k_0} \frac{I_0(k_0 h(x))}{I^2_1(k_0 h(x))}\partial _xI_1(k_0 h(x))\partial _x h(x). \end{aligned}$$

Hence,

78 $$\begin{aligned} B^\sigma _2(x) =&\frac{1}{2}B^\sigma _0(x)(\partial _x h(x))^2+\frac{I_0(k_0 h(x))}{k_0 I_1(k_0 h(x))}\partial _x B_0\partial _x h(x)\nonumber \\&+\frac{\partial _x^2 B_0 (x)}{2k_0^2} \left[ 1+k_0h(x)\frac{I_0(k_0h(x))}{I_1(k_0h(x))}\right] . \end{aligned}$$

Accordingly, the second-order correction to the integrated local charge can be obtained by substituting Eq. (78) into Eq. (70) and integrating along the radial direction (see “Appendix A”):

79 $$\begin{aligned} q^\sigma _2(x)&= -2\pi \epsilon k_0^2 \int _0^{h(x)} \phi _2(x,r)r dr\nonumber \\&= -\pi \sigma (\partial _x h(x))^2 -2\pi \epsilon I_0(k_0 h(x))h(x)\partial _x h(x)\partial _x B_0(x) \nonumber \\&\quad -2\pi \epsilon \frac{\partial _x^2 B_0 (x)}{2k_0^2} (k_0 h(x))^2 \left( I_0(k_0 h(x))-I_2(k_0 h(x))\right) \end{aligned}$$

Finally, recalling that, at second order, the surface charge per unit longitudinal length is

80 $$\begin{aligned} q^\sigma _w = 2\pi \sigma \left( 1+\frac{1}{2}(\partial _x h(x))^2\right) , \end{aligned}$$

the local charge excess, at second order in lubrication, reads (see “Appendix B”)

81 $$\begin{aligned} \Delta&q^\sigma (x)= -\partial _x \left[ \frac{\partial _x B_0 (x)}{\sigma k_0/\epsilon }h(x) I_1(k_0 h(x))\right] . \end{aligned}$$

**Fig. 6:**
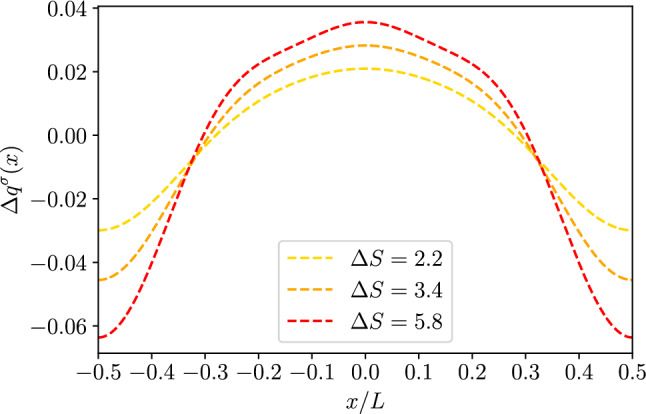
3*D* dielectric channel ($\sigma >0$). Local excess charge $\Delta q(x)$ as a function of the normalized position *x*/*L* along the channel axis for different values of $\Delta S$ as deailed in the legend with $h_0/L=0.1$ and $kh_0=1$

**Fig. 7:**
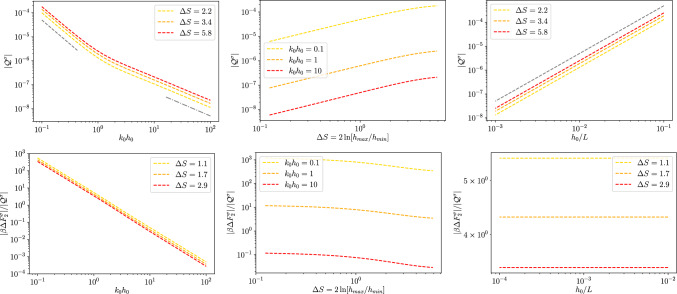
3*D* dielectric channel ($\sigma >0$). Top left: local excess charge $\Delta q^\sigma$ as a function of $k_0 h_0$ for different values of $\Delta S$ as reported in the legend and with $h_0/L=0.01$. The grey dashed line is proportional to $\propto (k_0 h_0)^{-1}$ and the dashed dotted to $\propto (k_0 h_0)^{-2}$. Top center: local excess charge $\Delta q^\sigma$ as a function of $\Delta S$ for different magnitudes of $kh_0$ as reported in the legend and with $h_0/L=0.1$. Top right: local excess charge $\Delta q^\sigma$ as a function of $h_0/L$ for different magnitudes of $\Delta S$ as reported in the legend and with $kh_0 = 1$. The thin grey dotted line is a guide for the eye and it is proportional to $(h_0/L)^2$. Bottom: ratio $\Delta F^\sigma _2/\mathcal {Q}^\sigma$ for the same values of the parameters of the corresponding top panel

Figure 6 shows the dependence of $\Delta q$ on the position. Interestingly, as for the 2*D* case, $\Delta q$ displays a maximum at the channel bottleneck, $x/L=0.5$. However, a part of the different shape of the profile, the main striking difference between Figs. 2 and 6 is the difference in magnitude of the effect. In fact, while for the 2*D* case the excess charge at the peak is comparable to the net charge density on the wall, for the 3*D* case (with similar geometry, $\Delta S$, and Debye length, $k_0 h_0$) the effect is weaker. Hence we do expect that local electroneutrality breakdown to be more prominent for slab-like channels then for cylindrical pores. More in detail, Fig. 7 shows again a similar trend as compared to the 2*D* case, Fig. 3, the only major difference being the non-monotonous dependence on $\Delta S$ shown for large values of $k_0 h_0$.

**Fig. 8:**
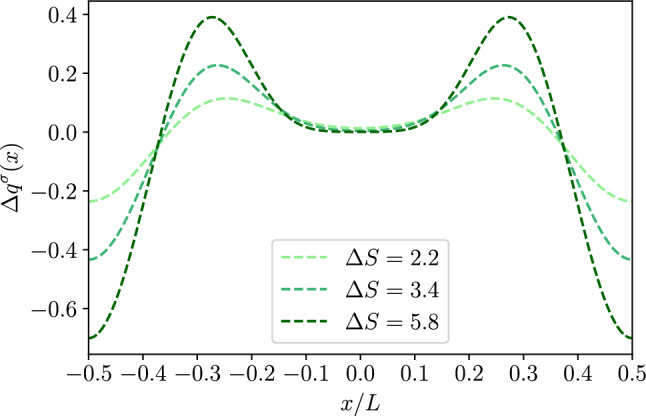
3*D* conducting channel ($\zeta >0$). Local excess charge $\Delta q(x)$ as a function of the normalized position *x*/*L* along the channel axis for different values of $\Delta S$ as detailed in the legend with $h_0/L=0.1$ and $kh_0=1$

*b. Conducting channel walls* For conducting channel walls the boundary condition is the same as the one derived for the 2*D* case, namely $\phi _2^\zeta (x,h(x))=0$, and hence Eq. (74) leads to

82 $$\begin{aligned} B^\zeta _2(x) =&\frac{\partial _x^2 B_0 (x)}{2k_0^2} \left[ 1+k_0h(x)\frac{I_1(k_0h(x))}{I_0(k_0h(x))}\right] \,. \end{aligned}$$

The second order correction to the wall charge can be obtained as in the 2*D* case, Eq. (56), where we change *y* for *r* and we multiply by the local area $2\pi h(x)(1+1/2(\partial _x h(x))^2)$:

83 $$\begin{aligned} q^\zeta _w =&-2\pi h(x)\epsilon \left[ I_0(k_0 h(x))\partial _xB_0^\zeta (x)\partial _x h(x)\right. \nonumber \\&-k_0\zeta \frac{I_1(k_0 h(x))}{I_0(k_0 h(x))}-B_2(x)k_0I_1(k_0 h(x))\nonumber \\&\left. +\frac{\partial _x^2B_0^\zeta (x)}{2k_0}\left( k_0 h(x)I_0(k_0 h(x))+I_1(k_0 h(x))\right) \right] \end{aligned}$$

Similarly to the 2*D* case, the net charge in the liquid phase reads

84 $$\begin{aligned} q_2^\zeta&= -2\pi \epsilon k_0^2 \int _0^{h(x)}\phi _2(x,r)r dr\nonumber \\&= -2\pi \epsilon k_0 h(x) \Big [B_2(x)I_1(k_0 h(x))\nonumber \\&\quad -\frac{1}{2k_0^2}\partial _x^2B_0(x)\left( I_1(k_0 h(x))+k_0 h(x) I_2(k_0 h(x))\right) \Big ]. \end{aligned}$$

**Fig. 9:**
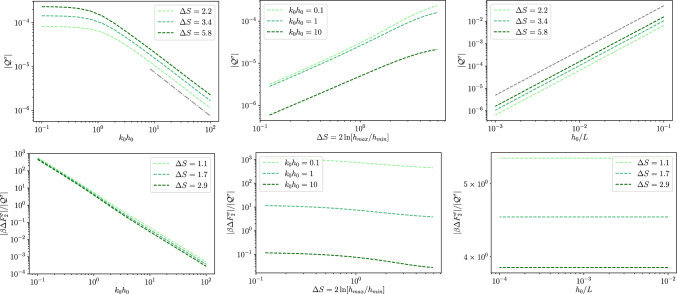
3*D* conducting channel ($\zeta >0$). Top: local excess charge $\Delta q^\sigma$ as a function of $k_0 h_0$ for different values of $\Delta S$ as reported in the legend and with $h_0/L=0.01$. The grey dashed line is proportional to $\propto (k_0 h_0)^{-1}$ and the dashed dotted to $\propto (k_0 h_0)^{-2}$. Center: local excess charge $\Delta q^\sigma$ as a function of $\Delta S$ for different magnitudes of $kh_0$ as reported in the legend and with $h_0/L=0.1$. Bottom: local excess charge $\Delta q^\sigma$ as a function of $h_0/L$ for different magnitudes of $\Delta S$ as reported in the legend and with $kh_0 = 1$. The thin grey dotted line is a guide for the eye and it is proportional to $(h_0/L)^2$. Bottom: ratio $\Delta F^\sigma _2/\mathcal {Q}^\zeta$ for the same values of the parameters of the corresponding top panel

Combining Eqs. (71), (83) and (84) leads to the local excess charge density

85 $$\begin{aligned} \Delta q^\zeta =-\frac{2}{k^2_0} \dfrac{\partial _x\left[ k_0h(x)I_1(k_0 h(x))\partial _x B_0^\zeta (x)\right] }{\zeta k_0 h_0 \frac{I_1(k_0h_0)}{I_0(k_0h_0)}}. \end{aligned}$$

as shown in Fig. 8,9

## 3*D* free energy barrier

Similarly to the 2*D* case, at equilibrium, the local free energy of a tracer ion with elementary charge *e* is given by

86 $$\begin{aligned} \beta F(x) = -\ln Z(x) = - \ln \left[ \frac{1}{\pi h^2_0}\int \limits _{0}^{h(x)}e^{-\beta e \phi (x,r)}rdr\right] \,. \end{aligned}$$

Within the Debye–Hückel approximation and expanding the logarithm, Eq. (62) can be decomposed into the following contributions:

87 $$\begin{aligned} \beta F_{DH}(x) \simeq \beta F_{gas}(x)+\beta F_{0}(x)+\beta F_{2}(x) , \end{aligned}$$

with

88 $$\begin{aligned} \beta F_{gas}(x)&= -2\ln \left[ \frac{h(x)}{h_0}\right] , \end{aligned}$$

89 $$\begin{aligned} \beta F_0(x)&= \frac{\beta e}{\pi h^2(x)}\int \limits _{-h(x)}^{h(x)}\phi _0(x,y)dy=-\frac{\beta e q_0(x)}{\pi \epsilon k_0^2 h^2(x)}, \end{aligned}$$

90 $$\begin{aligned} \beta F_2(x)&= \frac{\beta e}{\pi h^2(x)}\int \limits _{-h(x)}^{h(x)}\phi _2(x,y)dy=-\frac{\beta e q_2(x)}{\pi \epsilon k_0^2 h^2(x)}, \end{aligned}$$

where $\beta F_{gas}(x)$ is the free energy profile of an uncharged point particle whereas $\beta F_0(x)$ and $\beta F_2(x)$ are, respectively, the leading order and higher order correction for charged particles. In order to assess the impact of the local excess charge on the dynamics of a tracer ion, the bottom rows of Figs. 3 and 5 report the ratio between the second-order correction to the free energy difference

91 $$\begin{aligned} \Delta F_2 = F_2(L/2)-F_2(0) \end{aligned}$$

and the quadrupole moment. As already mentioned for the quadrupole, the overall behaviour of $\Delta F_2$ resembles the one observed in the respective 2*D* cases.

## Conclusions

In this contribution, we focus on the case of an electrolyte embedded between corrugated channel walls. In order to gain analytical insight we restrict our analysis to channels whose section is varying smoothly enough so that we can exploit the lubrication approximation to solve for the Poisson equation. Under such approximation, we have derived closed formulas for the corrections induced by the varying section of the channel to the local charge distribution. In particular, at equilibrium, the Debye length keeps homogeneous even when second-order corrections in the lubrication expansion are accounted for. At variance, while at first order in lubrication the local electroneutrality of the system is recovered, this is not so for second order corrections. This implies that upon reducing the length scale separation between the longitudinal and transverse direction the local excess charge will grow and this will also induce additional corrections to the effective free energy profile experienced by a tracer ion. Such local charge reorganization within corrugated channels has been observed so far only in out of equilibrium situations [28] where the advection of the ions plays a major role. Our results show that such a phenomenon occurs also at equilibrium and hence solely due to the interplay between the geometry of the channel and the electrostatic forces. In order to assess the robustness of our results we have derived such corrections for both dielectric and conducting channel walls in both planar (2*D*) and cylindrical (3*D*) geometries. Indeed, our results show quite a remarkable similarity between the 2*D* and 3*D* cases for both dielectric and conducting channel walls. Interestingly, the dielectric case shows an enhanced sensitivity to the dimensionality, as compared to the conducting case. In particular, the local (integrated) excess charge attains its maximum at the channel bottleneck ($x/L=0$), for 2*D* dielectric walls, and it can be as large as the bare charge (density) on the walls. This is not the case for cylindrical channels (3*D*) for which the local excess charge is $\simeq 100$ times smaller than the local charge. The difference in the location of the excess charge along the channel for dielectric and conducting walls indicates that the specific boundary conditions play a relevant role in the transport properties of confined electrolytes and ions. At variance, for conducting channels, the maxima of the local excess charge are located where the slope of the channel walls is maximum ($x/L=\pm 0.25$) for both 2*D* and 3*D* cases. All in all, the magnitude of the corrections that we report on are not very large and indeed this is expected since they are obtained via an expansion. However, this may not be the case when the longitudinal length is comparable to the Debye length. For typical values of the Debye length, $\lambda \simeq 10-100$nm, this implies to have channels with length $L\lesssim 100$nm. This is the case for many biological ionic channels as well as synthetic pores and membranes.

## Data Availability

The authors declare that the data supporting the findings of this study are available within the paper.

## Appendix A: Derivation of Eq. (48)

We want to show that

A1 $$\begin{aligned} \partial _x&\left[ \frac{\partial _x B_0 (x)}{2k_0^2}(k_0 h(x))^2 \left( I_0(k_0 h(x))-I_2(k_0 h(x))\right) \right] \nonumber \\&=- I_0(k_0 h(x))h(x)\partial _x h(x)\partial _x B_0(x) \nonumber \\&\quad - \frac{\partial _x^2 B_0 (x)}{2k_0^2} (k_0 h(x))^2 \left( I_1(k_0 h(x))-I_2(k_0 h(x))\right) \end{aligned}$$

that reduces to showing the following relation

A2 $$\begin{aligned} \partial _x&\left[ (k_0 h(x))^2 \left( I_0(k_0 h(x))-I_2(k_0 h(x))\right) \right] \nonumber \\ {}&=- 2I_0(k_0 h(x))h(x)\partial _x h(x) \end{aligned}$$

In order to do so we use the following relation

A3 $$\begin{aligned} \partial _x (x^n I_n(x))= x^n I_{n-1}(x) \end{aligned}$$

that leads to

A4 $$\begin{aligned} \partial _x\left[ (k_0 h(x))^2 I_2(k_0 h(x))\right] = (k_0 h(x))^2 I_1(k_0 h(x))\partial _x(k_0 h(x)) \end{aligned}$$

and

A5 $$\begin{aligned} \partial _x\left[ (k_0 h(x))^2 I_0(k_0 h(x))\right]&= 2 k_0 h(x) I_0(k_0 h(x)) \partial _x (k_0 h(x)) \nonumber \\&\quad + (k_0 h(x))^2 \partial _x I_0(k_0 h(x)))\nonumber \\&= 2k_0 h(x) I_0(k_0 h(x))\partial _x (k_0 h(x))\nonumber \\&\quad + (k_0 h(x))^2 I_1(k_0 h(x))\partial _x (k_0 h(x)) \end{aligned}$$

where in the last step we used

A6 $$\begin{aligned} I_{n+1}(x) = I_{n-1} - \frac{2n}{x}I_n(x) \end{aligned}$$

Summing Eqs. (A4), (A5) we get Eq. (A2) and hence Eq. (64).

## Appendix B: Derivation of Eqs. (79), (84)

In order to derive Eqs. (79), (84) we exploit the following relations

B1 $$\begin{aligned} \int _0^{h(x)} I_0 (k_0 r) r dr&= \frac{1}{k_0^2}\int _0^{k_0 h(x)} I_0 (z) z dz \nonumber \\ {}&= \frac{1}{k_0^2}k_0 h(x) I_1 (k_0 h(x)) \end{aligned}$$

B2 $$\begin{aligned} \int _0^{h(x)} k_0 r I_1(k_0 r) r dr&= \frac{1}{k_0^2}\int _0^{k_0 h(x)} I_1 (z) z^2 dz \nonumber \\ {}&= \frac{1}{k_0^2}(k_0 h(x))^2 I_2 (k_0 h(x)) \end{aligned}$$

were we used $z=k_0 r$. Finally we get:

B3 $$\begin{aligned} q_2(x)&= -2\pi \epsilon k_0^2 \int _0^{h(x)}\phi _2(x,r)r dr\nonumber \\&= -2\pi \epsilon k_0 h(x) \left[ B_2(x)I_1(k_0 h(x))\right. \nonumber \\&\quad \left. -\frac{1}{2k_0^2}\partial _x^2B_0(x)\left( I_1(k_0 h(x))+k_0 h(x) I_2(k_0 h(x))\right) \right] \end{aligned}$$

By substituting the relative expressions for $B_2(x)$ and $B_0(x)$ we get

B4 $$\begin{aligned} q_2^\sigma (x)&= -\pi \sigma h(x) -2\pi \epsilon I_0(k_0 h(x))h(x)\partial _x h(x)\partial _x B_0(x) \nonumber \\&\quad -2\pi \epsilon \frac{\partial _x^2 B_0 (x)}{2k_0^2} (k_0 h(x))^2 \left( I_0(k_0 h(x))-I_2(k_0 h(x))\right) \end{aligned}$$

B5 $$\begin{aligned} q_2^\zeta (x)&= -2\pi \epsilon \frac{\partial _x^2 B_0(x)}{2k_0^2}\left( k_0 h(x)\right) ^2\left[ \frac{I_1^2(k_0 h(x))}{I_0(k_0 h(x))}-I_2(k_0 h(x))\right] \end{aligned}$$

Finally, using the recursive relation

B6 $$\begin{aligned} \partial _x I_0(k_0 h(x))= I_1(k_0 h(x))k_0 \partial _x h(x) \end{aligned}$$

we obtain

B7 $$\begin{aligned} q_2^\sigma (x)&= -\pi \sigma h(x) -2\pi \epsilon I_0(k_0 h(x))h(x)\partial _x h(x)\partial _x B_0(x) \nonumber \\&\quad +2\pi \epsilon \frac{\partial _x^2 B_0 (x)}{k_0^2} k_0 h(x) I_1(k_0 h(x)) \end{aligned}$$

## Footnotes

1 We note that, at zeroth order in lubrication, the surface charge at each conducting wall can be obtained by $\sigma ^\zeta =\pm \epsilon \partial _y\phi _0^\zeta (x,y)|_{y=\pm h(x)}=\epsilon k_0 \zeta \tanh (k_0 h(x))$.

2 We recall that $\int _{-h(x)}^{h(x)} k^2y \sinh (ky)dy = 2\,h k \cosh (h k) - 2 \sinh (h k)$.

3 We recall that $\int _{-h(x)}^{h(x)} k^2y \sinh (ky)dy = 2\,h k \cosh (h k) - 2 \sinh (h k)$

4 We recall the following property of the modified Bessel function of the first kind $\partial _z(z^n I_n(z))=z^n I_{n-1}(z)$
